# A Knowledge-Based System for Display and Prediction of *O*-Glycosylation Network Behaviour in Response to Enzyme Knockouts

**DOI:** 10.1371/journal.pcbi.1004844

**Published:** 2016-04-07

**Authors:** Andrew G. McDonald, Keith F. Tipton, Gavin P. Davey

**Affiliations:** School of Biochemistry and Immunology, Trinity College Dublin, Dublin, Ireland; University of California San Diego, UNITED STATES

## Abstract

O-linked glycosylation is an important post-translational modification of mucin-type protein, changes to which are important biomarkers of cancer. For this study of the enzymes of *O*-glycosylation, we developed a shorthand notation for representing GalNAc-linked oligosaccharides, a method for their graphical interpretation, and a pattern-matching algorithm that generates networks of enzyme-catalysed reactions. Software for generating glycans from the enzyme activities is presented, and is also available online. The degree distributions of the resulting enzyme-reaction networks were found to be Poisson in nature. Simple graph-theoretic measures were used to characterise the resulting reaction networks. From a study of *in-silico* single-enzyme knockouts of each of 25 enzymes known to be involved in mucin *O*-glycan biosynthesis, six of them, *β*-1,4-galactosyltransferase (*β*4Gal-T4), four glycosyltransferases and one sulfotransferase, play the dominant role in determining *O*-glycan heterogeneity. In the absence of *β*4Gal-T4, all Lewis X, sialyl-Lewis X, Lewis Y and Sd^a^/Cad glycoforms were eliminated, in contrast to knockouts of the *N*-acetylglucosaminyltransferases, which did not affect the relative abundances of *O*-glycans expressing these epitopes. A set of 244 experimentally determined mucin-type *O*-glycans obtained from the literature was used to validate the method, which was able to predict up to 98% of the most common structures obtained from human and engineered CHO cell glycoforms.

## Author Summary

Our objective being to model the enzymes of mucin-type O-linked glycosylation, we first developed a model language to represent *O*-glycan structures succinctly in linear string form, to which a set of pattern-matching rules was then applied to simulate the activities of a set of 25 glycosyltransferase and sulfotransferase enzymes. The modelling language (a formal language), together with the set of transformation rules representing the enzymes of the model. comprise the deductive apparatus of a formal system. The system, implemented in software, was able to predict a highly heterogeneous set of structures when all enzymes were allowed to act, including many clinically important epitopes such as sialyl-Lewis X. We studied the effects of single-enzyme knockouts on the properties of the resulting enzyme-catalysed reaction networks and determined the enzymes most likely to be responsible for heterogeneity.

## Introduction

Glycosylation is a major post-translational modification of proteins, in which a carbohydrate moiety, called a glycan, is covalently attached to an amino acid of the polypeptide, to form a glycoprotein [1]. N-linked glycans are attached to an asparagine (N) residue appearing in the consensus sequence Asn-X-Ser/Thr, where X is not Pro, while O-linked glycans are attached to the hydroxyl group of a serine or threonine residue. A study of potential glycosylation sites indicated that three quarters of proteins may be glycosylated, with about 10% of these *O*-glycosylated [2]. Glycans are formed by the sequential addition of monosaccharides from nucleotide-sugar donors to the glycoprotein acceptor, a process that is catalysed by glycosyltransferase enzymes, which are located in the endoplasmic reticulum and Golgi apparatus.

Mucins are a class of large glycoproteins that contain a large number of Ser/Thr in close proximity, which can be heavily *O*-glycosylated. The initial step of mucin-type glycosylation is the attachment of a GalNAc (*N*-acetyl-d-galactosamine) to an unoccupied Ser/Thr on the protein acceptor. Modification of mucin *O*-glycosylation is an important biomarker in cancer detection [3–8]. In the innate immune response, cell-cell recognition is dependent on the expression of a number of different carbohydrate epitopes on carrier proteins, which include both sulfated and non-sulfated versions of Lewis X (Le^x^), Lewis A (Le^a^), Lewis B (Le^b^) [9] and, more rarely, Lewis Y (Le^y^) antigens [10].

Of the several theoretical treatments of glycosylation which have now appeared, most have considered *N*-glycosylation rather than *O*-glycosylation [11]. The method of Kawano *et al.* [12] for predicting glycan structures from gene expression data was able to predict the appearance of a variety of glycosylated structures, including O-linked. The model by Gerken and co-workers focused on the initiation of *O*-glycosylation [13]. Liu *et al.* [14] described an object-oriented method of construction of networks of *O*-glycan biosynthesis that was used to predict levels of sialyl-Lewis X (SLe^x^), an important antigenic determinant, and more recently a computational approach based on MATLAB has been used to predict pathways of N- and O-linked glycosylation [15, 16]. In the present work, we have taken an alternative, bottom-up, approach to modelling the *de novo* biosynthesis of mucin *O*-glycans. In order to facilitate computational analysis, we introduce a formal language (see [17]) for identifying individual glycan structures, a method for representing glycans graphically, based on these identifiers, and describe a method for generating networks of reactions based on the activities of enzymes involved in mucin protein *O*-glycosylation. A mathematical model of N-linked glycosylation has been developed, [18] whose structure identifiers are based on Linear Code; Spahn *et al.* have developed a Markov-chain model based on this system. [19]. As it seeks to uncover the nature of the reaction networks of *O*-glycosylation, this work both validates and extends the approach used by these earlier studies.

With a rapidly increasing number of studies employing nuclease-based genome-editing technologies, such as zinc-finger nuclease (ZFN) [20] and CRISPR/Cas9 [21], for biotechnological applications, it is important to consider the possible phenotypic effects that may result from knock-ins or knockouts of the glycosyltransferase genes, and the corresponding changes to the glycome. We anticipate that the methods we describe here will be of use in predicting such changes within the context of *O*-glycosylation networks.

## Methods

A study of the GalNAc-linked oligosaccharides within the online repository of the Consortium for Functional Glycomics [22] revealed the five most commonly occurring monosaccharides to be d-galactose (Gal), *N*-acetylgalactosamine (GalNAc), *N*-acetylglucosamine (GlcNAc), l-fucose (Fuc) and *N*-acetylneuraminic acid (Neu5Ac). The five most commonly encountered sugars were: Gal (32.3%), GalNAc (22.7%), GlcNAc (20.7%), Fuc (11.2%) and Neu5Ac (9.6%). Four residues, which included *N*-glycolylneuraminic acid (Neu5Gc) and 2-keto-3-deoxy-d-*glycero*-d-*galacto*-nononic acid (Kdn), made up the remaining 4% of the total monosaccharide composition. Methylated and sulfated variants were included in the analysis.

At the time of writing, 1654 transferases are listed in the IUBMB Enzyme Nomenclature, of which 280 involve the transfer of a monosaccharide from a nucleotide-sugar donor to an acceptor. An examination of the latter subset of reactions reveals that the class of monosaccharides employed is quite small, with over 90% of the glycosyltransferase reactions involving only 8 distinct sugar species, Fuc, Gal, GlcA, GalNAc, Glc, GlcNAc, Neu5Ac and Xyl. Combined with the result of the analysis of the CFG database, this suggested that the language of *O*-glycosylation has a limited alphabet, though with a potentially rich vocabulary. A formal language was developed that uses a single-letter code for the five most commonly encountered monosaccharides, with uppercase letters for d-sugars and lowercase for the less common l isomers. The symbols of the language and their meanings are summarised in Table 1.

**Table 1:** Symbols used in *O*-glycan identifiers.

Symbol	IUPAC Symbol	Definition
f	Fuc	l-Fucose
K	Kdn	2-Keto-3-deoxy-d-*glycero*-d-*galacto*-nononic acid
L	Gal	d-Galactose
N	Neu5Gc	*N*-Glycolylneuraminic acid
S	Neu5Ac	*N*-Acetylneuraminic acid (sialic acid)
V	GalNAc	*N*-Acetyl-d-galactosamine
Y	GlcNAc	*N*-Acetyl-d-glucosamine
s	-SO3H	Sulfate
a, b	*α*, *β*	Anomeric configuration
[,]	[,]	Branch delimiters
T		Protein backbone

**Fig 1:**
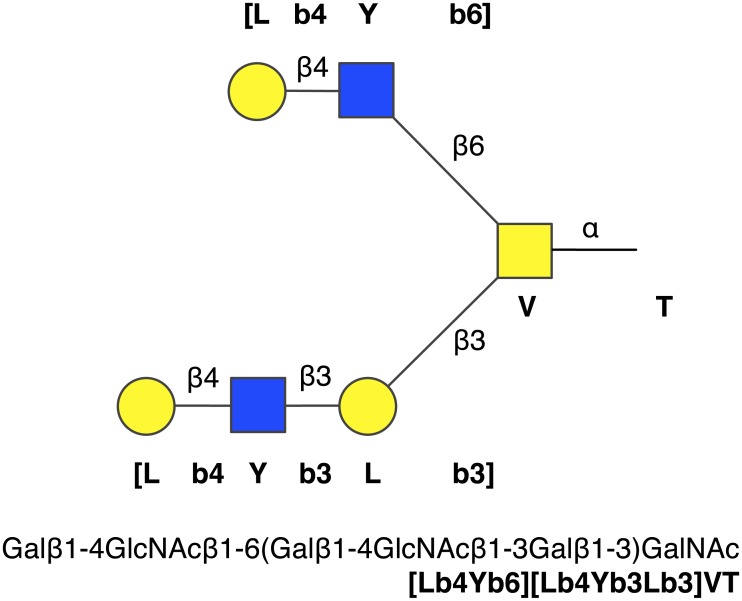
Structure identifier example. The diantennary *O*-glycan defined by the structure identifier [Lb4Yb6][Lb4Yb3Lb3]VT, with its IUPAC name in linear condensed form.

The strings generated by the language, which we refer to as *structure identifiers*, are a further contraction of the short-form, one-line representation of oligosaccharides [23], in which the IUPAC sugar symbols are replaced by one-letter codes, and brackets instead of parentheses are used as branch delimiters. An example *O*-glycan is shown in Fig 1.

We identified 25 distinct enzyme activities in which these common monosaccharides are transferred during GalNAc-linked glycosylation, which are shown in Table 2. The *O*-glycan structure indentifiers enable us to write the reactions catalysed by these enzymes more succinctly. For instance, the ST3Gal-I reaction, CMP-*N*-acetylneuraminate + *N*-acetyl-*α*-neuraminyl-(2 → 3)-*β*-d-galactosyl-(1 → 3)-*N*-acetyl-d-galactosaminyl-R = CMP + *N*-acetyl-*α*-neuraminyl-(2 → 3)-*β*-d-galactosyl-(1 → 3)-[*N*-acetyl-*α*-neuraminyl-(2 → 6)]-*N*-acetyl-d-galactosaminyl-R can be represented in the current notation as

$$\begin{array}{c} \text{CMP}\;-\;S+[\text{Sa}3\text{Lb}3]\text{VT}=\text{CMP}+[\text{Sa}6][\text{Sa}3\text{Lb}3]\text{VT} \end{array}$$

where CMP-S is the donor and [Sa3Lb3]VT is the acceptor. Table 2 shows the enzyme reactions using a shorthand form based on the formal language. For simplicity, the stereochemical information (a/b) will be omitted within the text, based on the known specificities of the enzymes. For the enzymes considered in this model, all of the fucosyltransferases and sialyltransferases produce *α*-linked structures. The galactosyltransferases and *N*-acetylglucosaminyltransferases will be assumed to form *β*-linked products, unless indicated otherwise, while *N*-acetylgalactosaminyltransferases will be assumed to form *α* products. Hence, without ambiguity, we can rewrite the reaction equation above as

$$\begin{array}{c} \text{CMP}\;-\;S+[S3L3]\text{VT}=\text{CMP}+[S6][S3L3]\text{VT} \end{array}$$

**Table 2:** The enzymes of *O*-glycosylation included in this study.

	Abbreviation	EC Number	IUBMB Name	Reaction
**1**	*β*4Gal-T4	EC 2.4.1.38	*β*-*N* -acetylglucosaminylglycopeptide	UDP-L + *[Y*T = UDP + *[Lb4Y*T
			*β*-1,4-galactosyltransferase	
**2**	ppGalNAc-T	EC 2.4.1.41	polypeptide *N* -acetylgalactosaminyltransferase	UDP-V + T = UDP + VT
**3**	*α*4Fuc-T	EC 2.4.1.65	3-galactosyl-*N* -acetylglucosaminide	GDP-f + *[Lb3Y*T = GDP + *[Lb3[fa4]Y*T
			4-*α*-l -fucosyltransferase	
**4**	*α*2Fuc-Ts	EC 2.4.1.69	galactoside 2-*α*-l -fucosyltransferase	GDP-f + *[Lb3Y*T = GDP + *[[fa2]Lb3Y*T
				GDP-f + *[Lb3]VT = GDP + *[[fa2]Lb3]VT
**5**	C2Gn-T	EC 2.4.1.102	*β*-1,3-galactosyl-*O* -glycosyl-glycoprotein	UDP-Y + [Lb3]VT = UDP + [Yb6][Lb3]VT
			*β*-1,6-*N* -acetylglucosaminyltransferase	
**6**	C1Gal-T1	EC 2.4.1.122	glycoprotein-*N* -acetylgalactosamine	UDP-L + VT = UDP + [Lb3]VT
			3-*β*-galactosyltransferase	
**7**	*β*3Gn-T3	EC 2.4.1.146	*β*-1,3-galactosyl-*O* -glycosyl-glycoprotein	UDP-Y + [Yb6][Lb3]VT = UDP + [Yb6][Yb3Lb3]VT
			*β*-1,3-*N* -acetylglucosaminyltransferase	
**8**	*β*3Gn-T6	EC 2.4.1.147	acetylgalactosaminyl-*O* -glycosyl-glycoprotein	UDP-Y + VT = UDP + [Yb3]VT
			*β*-1,3-*N* -acetylglucosaminyltransferase	
**9**	C2/4Gn-T	EC 2.4.1.148	acetylgalactosaminyl-*O* -glycosyl-glycoprotein	UDP-Y + [Yb3]VT = UDP + [Yb6][Yb3]VT
			*β*-1,6-*N* -acetylglucosaminyltransferase	
**10**	*β*3Gn-T2/3/4/5/7	EC 2.4.1.149	*N* -acetyllactosaminide *β*-1,3- *N* -acetyl-	UDP-Y + *[Lb4Y*T = UDP + *[Yb3Lb4Y*T
			glucosaminyltransferase	
**11**	*α*3Fuc-T	EC 2.4.1.152	4-galactosyl-*N* -acetylglucosaminide	GDP-f + *[Lb4Y*T = GDP + *[Lb4[fa3]Y*T
			3-*α*-l-fucosyltransferase	
**12**	*β*3Gal-T5	EC 2.4.1.-	(*β*-*N* -acetylglucosaminylglycopeptide	UDP-L + *[Y*T = UDP + *[Lb3Y*T
			*β*-1,3-galactosyltransferase)	
**13**	ST6Gal-I	EC 2.4.99.1	*β*-galactoside *α*-2,6-sialyltransferase	CMP-S + *[Lb4Y*T = CMP + *[Sa6Lb4Y*T
**14**	ST6GalNAc-I	EC 2.4.99.3	*α*-*N* -acetylgalactosaminide *α*-2,6-sialyl-	CMP-S + VT = CMP + [Sa6]VT
			transferase	CMP-S + [Lb3]VT = CMP + [Sa6][Lb3]VT
**15**	ST3Gal-I	EC 2.4.99.4	*β*-galactoside *α*-2,3-sialyltransferase	CMP-S + *[Lb3]VT = CMP + *[Sa3Lb3]VT
**16**	ST3Gal-III/IV	EC 2.4.99.6	*N* -acetyllactosaminide *α*-2,3-sialyltransferase	CMP-S + *[Lb4Y*T = CMP + *[Sa3Lb4Y*T^a^
**17**	ST6GalNAc-III/IV	EC 2.4.99.7	*α*-*N* -acetylneuraminyl-2,3-*β*-galactosyl-1,3- *N*-	CMP-S + [Sa3Lb3]VT = CMP + [Sa6][Sa3Lb3]VT
			acetylgalactosaminide 6-*α*-sialyltransferase	
**18**	ST6GlcNAc-I	EC 2.4.99.-	(*α*-*N* -acetylneuraminyl-2,3-*β*-galactosyl-1,4-	CMP-S + *[Yb3Lb3]VT = CMP + *[Sa6Yb3Lb3]VT
			*N* -acetylgalactosaminide 6-*α*-sialyltransferase)	
**19**	Gcnt2 (I-GnT)	EC 2.4.1.-	(*N* -acetyllactosaminide *β*-1,6- *N* -acetyl-	UDP-Y + *[Lb4Yb3L*T = UDP + *[[Yb6][Lb4Yb3]L*T
			glucosaminyltransferase)	
**20**	CHST4/6	EC 2.8.2.-	(GlcNAc-6-*O* -sulfotransferase)	PAP-s + *[Y*T = ABP + *[[s6]Y*T^b^
**21**	GAL3ST2	EC 2.8.2.-	(*β*1,3-Gal 3-*O* -sulfotransferase)	PAP-s+ *[Lb3*T = ABP + *[[s3]Lb3*T^b^
**22**	GAL4ST4	EC 2.8.2.-	(*β*1,4-Gal 3-*O* -sulfotransferase)	PAP-s+ *[Lb4*T = ABP + *[[s3]Lb4*T^b^
**23**	*α*3Gal-T	EC 2.4.1.37	(*N* -acetyllactosaminide *β*-1,6- *N* -acetyl-	UDP-L + *[[fa2]L*T = UDP + *[La3[fa2]L*T
			glucosaminyltransferase)	
**24**	*α*3GalNAc-T	EC 2.4.1.40	glycoprotein-fucosylgalactoside *α*-*N* -acetyl-	UDP-V + *[[fa2]L*T = UDP + *[Va3[fa2]L*T
			galactosaminyltransferase	
**25**	*β*4GalNAc-T	EC 2.4.1.-	(glycoprotein-sialylgalactoside *β*-1,4-*N* -acetyl-	UDP-V + *[Sa3Lb4*T = UDP + *[Sa3[Vb4]Lb4*T
			galactosaminyltransferase)	

Abbreviated forms of enzyme reaction equations, including anomeric linkage types *α*/*β* (a/b). Where an EC number is unavailable, the expected sub-subclass is given. T denotes a Ser/Thr *O* -glycosylation site on the protein. An asterisk symbol acts as a wildcard character, denoting an oligosaccharide of unspecified length. Abbreviations used: PAP-s, 3′-phosphoadenosine-5′-phosphosulfate (PAPS); ABP, adenosine 3′,5′-bisphosphate; other symbols are defined in the text.

^a^Can also act on type-1 acceptors.

^b^The products of sulfotransferase action (enzymes **20**–**22**) do not block the activities of the other transferases.

A consequence of the formal grammar is that any residue added to the base GalNAc is treated as a branch. Therefore [L3]VT is written instead of L3VT, and [S6][S3L3]VT instead of S3L3[S6]VT. While we could write [Y3[Y6]L4Y3]VT to represent GlcNAc*β*1-3(GlcNAc*β*1-6)Gal*β*1-4GlcNAc*β*1-3GalNAc, by convention we will write such structures as [[Y6][Y3]L4Y3]VT, even though both are valid according to the grammar. Branches at the same level are written from right to left in ascending linkage order, as shown in Table 2.

### Structure identifiers defined by a formal grammar

We introduce a formal grammar [24], Γ = (Σ_N_, Σ_T_, **P**, **S**), where Σ_N_ is a set of *nonterminal* symbols and Σ_T_ is a set of *terminal* symbols. Σ_N_ and Σ_T_ are disjoint sets, meaning that they share no members in common. **S** defines a starting symbol and **P** is a set of production rules, each element of which maps a single non-terminal symbol to a string of one or more symbols drawn from Σ_T_∪Σ_N_, or to the null (empty) string, *ϵ*.

ΣN={Z,A,B,C,m,d,l}ΣT={2,3,4,6,8,a,b,f,s,K,L,N,S,T,V,Y,[,]}P=Z→ATA→ϵ|BBVB→ϵ|[Cmld]C→ϵ|Cmld|C[Cmld]m→f|s|K|L|N|S|V|Yd→2|3|4|6|8l→ϵ|a|bS=Z

The grammar generates a language L by the successive substitution of nonterminal symbols with the right-hand sides of production rules in **P**. The set Σ_T_ ∪ Σ_N_ is the alphabet of L, and strings of symbols generated by Γ are the *words* of the language. We define a *structure identifier* as a word of L that contains only symbols drawn from Σ_T_.

The following sequence of strings serves as an example of a derivation within the grammar. For brevity, some steps are the result of several simultaneous applications of production rules.

$$\begin{array}{cc} Z\\ A\text{T} & \left\{Z\to A\text{T}\right\}\\ BB\text{VT} & \left\{A\to BB\text{V}\right\}\\ {}[Cmld][Cmld]\text{VT} & \left\{B\to [Cmld]\right\}\\ {}[Cmld][mldmld]\text{VT} & \left\{C\to Cmld,C\to \epsilon \right\}\\ {}[mld][mldmld]\text{VT} & \left\{C\to \epsilon \right\}\\ {}[\text{S}ld][\text{S}ld\text{L}ld]\text{VT} & \left\{m\to \text{S},m\to \text{L}\right\}\\ {}[\text{S}6][\text{S}3\text{L}3]\text{VT} & \left\{d\to 6,d\to 3,l\to \epsilon \right\}\\ \; \end{array}$$

The final string in the list is a word in Γ denoting disialylated T antigen, commonly known as “diST”, a core-1 *O*-glycan.

#### Interpretation of the formal grammar

We give the following interpretation for the language generated by Γ. We let the terminal symbol T represent a protein backbone, or, more specifically, either a serine or threonine. The nonterminal symbol *m* represents either (1) a member of the set of monosaccharide one-letter codes {f,K,L,N,S,V,Y}, which in turn correspond to the monosaccharides l-fucose (Fuc), 2-keto-3-deoxy-d-*glycero*-d-*galacto*-nononic acid (Kdn), d-galactose (Gal), *N*-glycolylneuraminic acid (Neu5Gc), *N*-acetylneuraminic acid (Neu5Ac), *N*-acetylgalactosamine (GalNAc) and *N*-acetylglucosamine (GlcNAc) or (2) a modifier symbol in {s}, which represents sulfate. The one-letter code is based in part upon that of the GLYCAM system [25], which uses lowercase letters to represent l-sugars and uppercase letters for d-sugars, except that we use the single letter ‘S’ to denote *N*-acetylneuraminic acid. Since, to our knowledge, an l-variant of *N*-acetylneuraminate is unknown to *O*-glycosylation, a lowercase ‘s’ has been used to represent sulfate (-SO3H). The nonterminal symbol *d* denotes the linkage position on the parent sugar residue, while *l* represents the linkage type. The terminal symbols ‘a’ and ‘b’ denote the *α* and *β* anomers, respectively. *O*-Glycan branches are enclosed within matching pairs of brackets. In the context of the present work, only the linkage positions 2, 3, 4 and 6 of hexose sugars are used.

With the introduction of a deductive system that allows certain strings to be derived from others, the question arises as to whether the language L is preserved by the transformations given in Table 2. The outline of a proof that the language is preserved by the reaction schemata is as follows.

**Theorem.** The language L is preserved by the reaction schemata of Table 2.

*Proof.* The reaction schemata can be divided into two classes that depend on the absence or presence of the wildcard character, *. For each acceptor substrate and product of the enzyme reactions of Table 2 in which * does not appear, derive the corresponding structure identifier in Γ starting from the initial letter, *Z*. Otherwise, proceed as follows. Let *xWy* be a word in L, where *W* ∈ {*A*T,[*C*,*CL*,[*CY*} and *x* and *y* are word fragments, and *x*, but not *y*, can be the null string. *W* is the minimum set of strings required to derive all of the pattern-based enzyme rules in Table 2, each element of which is based upon the right hand sides of one or more production rules. Apply the production rules to an element of *W* to derive the sub-structure identifier, *W*′, corresponding to that class of substrate or product. Since xWy∈L, then $xW^{\prime }y\in L$ also.

Each case is illustrated by an example.

Case 1. Ignoring donor molecules, reaction **8** can be written as VT → [Yb3]VT. The derivations of substrate and product are

$$\begin{array}{cc} Z & \\ AT & \left\{Z\to AT\right\}\\ \text{VT} & \left\{A\to V\right\} \end{array}$$

and

$$\begin{array}{cc} Z & \\ AT & \left\{Z\to AT\right\}\\ B\text{VT} & \left\{A\to BV\right\}\\ {}[mld]\text{VT} & \left\{B\to [mld]\right\}\\ {}[\text{Yb}3]\text{VT} & \left\{m\to Y,l\to b,d\to 3\right\} \end{array}$$

where the production rules used are shown to the right of each step. Therefore, VT and [Sa6]VT are both members of L.

Case 2. Reaction **11** involves a pattern, and can be written as *[Lb4Y*T → *[Lb4[fa3]Y*T. Let *xWy* be a word in L where *W* = [*CY*. The corresponding derivations are

$$\begin{array}{cc} {}[C\text{Y} & \\ {}[mld\text{Y} & \left\{C\to mld\right\}\\ {}[\text{Lb}4\text{Y} & \left\{m\to L,l\to b,d\to 4\right\} \end{array}$$

and

$$\begin{array}{cc} {}[C\text{Y} & \\ {}[C[mld]\text{Y} & \left\{C\to C[mld]\right\}\\ {}[mld[mld]\text{Y} & \left\{C\to mld\right\}\\ {}[\text{Lb}4[\text{fa}3]\text{Y} & \left\{m\to L,l\to b,d\to 4,m\to f,l\to a,d\to 3\right\} \end{array}$$

Since xWy∈L, *x*[Lb4Y*y* and *x*[Lb4[fa3]Y*y* are also words in L. The remainder of the proof follows by similar reasoning for each of the other reactions, the details of which are left to the reader.

### Software

The linear string identifiers described in this work can be used to draw glycan structures in the manner of turtle graphics [26]. Reading the identifier from right to left, the drawing method acts according to the current symbol: if the symbol is an element of the set {f,K,L,N,S,V,Y,s}, it draws the symbol corresponding to the monosaccharide at the current drawing position; if the string character is a right bracket,], the current position and orientation information are pushed onto a stack, and are popped from the stack on meeting a left bracket. A two-pass approach is employed, with the bond framework being drawn on the first pass, and the sugar symbols drawn on the second.

A suite of Perl scripts was written for the generation of structure identifiers by enzyme simulation, for parsing, and rendering as Scalable Vector Graphics (SVG) image files. A library of functions was written as a Perl module, which enabled (i) the translation of structure identifiers to and from the IUPAC condensed-form one-line notation; (ii) identification of common epitopes, such as Le^x^, based on regular-expression patterns; (iii) parsing of *O*-glycan strings by an LL(1) parser based on a simplified version of Γ; (iv) rendering of string identifiers as SVG, in either UOXF or CFG styles.

#### O-Glycologue

A web application was written to draw *O*-glycan structures based on strings entered by the user; called O-Glycologue, it is a significant upgrade to the original [27], which was designed to draw *N*-glycan structures based on a nine-digit code formalism described by Krambeck and Betenbaugh [28]. The new version (available at http://www.boxer.tcd.ie/glycologue) is able to display structures entered by the user in either the one-line IUPAC condensed form, or the shortened notation described in this work, and to submit these to the enzyme simulator. The set of graphical symbols used is based upon that of the Consortium for Functional Glycomics (CFG) [29] but support for Oxford (UOXF) [30] symbolism is also provided. Linkage positions are interpreted according to the desired output style. Sulfated residues are indicated by a small orange star on the upper-left (6-sulfation) or lower-left (3-sulfation) of the monosaccharide, or by a lowercase ‘s’ when UOXF symbols were selected.

Once drawn, the image can be saved as Scalable Vector Graphics, or redrawn in an alternative symbolism (CFG or UOXF). In addition to accepting IUPAC names as input, the application also displays the IUPAC condensed linear form, Linear Code [31] and condensed GlycoCT [32] representations beneath the current structure, which can then be imported into other glycoinformatics tools, such as GlycoWorkbench [33]. The control panel at the upper left of the browser window is used to select the number of iterations used by O-Glycologue, and to place a limit on the number of GlcNAc residues incorporated into glycans. If the prediction tool is selected, the string is submitted as a substrate to the enzymes of *O*-glycosylation acting in reverse, until ppGalNAc-T has removed GalNAc from the protein or no further products have been formed after the current iteration. The current structure can be submitted to the enzyme simulator as a starting substrate, which will generate all of the possible *O*-glycan products as a table. The web application can be adjusted to use only a user-selected set of enzymes by selecting the appropriate menu option, which lists the enzymes involved, and marking each with a checkbox that can be used to knock out its activity.

With all of the enzymes of Table 2 active, the method will generate 8,930 unique *O*-glycans in 8 iterations, when starting from an non-glycosylated protein site and with no limit placed on the number of GlcNAcs incorporated. Knockouts can be compared with the full set of glycans by selecting the appropriate option beneath the list of enzymes. Any set of knockouts can be set as a new baseline against which the effects of additional knockouts can be compared. When comparing with the baseline, O-Glycologue runs the simulation twice, once with all enzymes active, and the second time with the selected enzymes disabled, leaving the missing structures as gaps in the table. The display of the missing structures from the full set of glycans can be toggled. Structure identifiers are printed beneath each *O*-glycan, by default, but can be hidden. Each structure links to GlycoForm, from where it can be exported as an image file or submitted as a substrate to O-Glycologue. The numbers of structures of each core type (1–4) [34] and those of common antigenic epitopes, such as Lewis A, B, X and Y, are printed after the table of *in-silico* generated *O*-glycans. For the example above, after 8 iterations of the method, 1,536 *O*-glycans were found to be of Core-1 type (Gal*β*1-3GalNAc-Ser/Thr), 2,828 were Core 2 (GlcNAc*β*1-6[Gal*β*1-3]GalNAc-Ser/Thr), 1,011 were Core 3 (GlcNAc*β*1-3GalNAc-Ser/Thr) and 3,553 were Core 4 (GlcNAc*β*1-6[GlcNAc*β*1-3]GalNAc-Ser/Thr). The two remaining structures that were outside this classification were the tumour-associated antigens Tn (GalNAc-Ser/Thr) and Sialyl-Tn ([Neu5Ac*α*2-3]GalNAc-Ser/Thr).

To minimise page build times in O-Glycologue, glycan images are prerendered and saved as PNG files. If a glycan image is not found, it is generated automatically and stored on the server for future use. At higher iterations, the task of laying out reaction networks becomes prohibitive in terms of execution time. For this reason, networks that are larger than 5,000 nodes are not rendered with GraphViz but are instead provided as downloadable DOT files. Reaction networks can be downloaded as SBML Level 2 (version 4) for use in other applications.

## Results

### Enzyme reaction simulations

**Fig 2:**
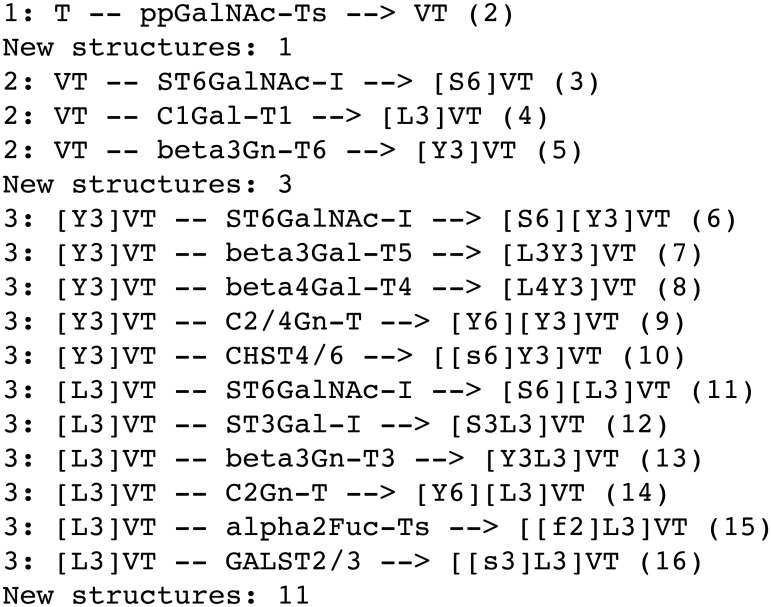
Enzyme simulation. Output of the Perl script used to mimic the actions of the enzymes of Table 2, for four iterations of the method described in the text. Each *in-silico* reaction takes the form <iteration no.>: <substrate> -- <enzyme> --> <product> (<serial no.>). Each new product is assigned a serial number, the value of which is incremented by one at the appearance of each new *O*-glycan.

Not all of the structures encoded by the formal grammar are feasible, in that structures such as [S3][L3]VT are syntactically correct, but chemically impossible, since it describes a sialic acid (S) and galactose (L) both 3-linked to the same *N*-acetylgalactosamine (V). In order to generate a set of biologically relevant *O*-glycans, therefore, a set of regular-expression based substitution rules was developed to mimic the actions of each of the enzymes shown in Table 2; throughout this work, numbers in bold face refer to the corresponding activities in this table. The rules were incorporated into a Perl script, which took a single *O*-glycan identifier as the initial substrate, and applied each of the substitutions in turn to output a set of products. The initial structure defaulted to the non-glycosylated site, ‘T’, but any valid glycan structure could be supplied by the user as a starting point. The process was applied iteratively, such that each new product formed was presented as a substrate to every enzyme upon the next iteration. Where an enzyme rule could match at more than one position, as in the case of diantennary *O*-glycans, the identifier was split, using the current regular expression, and then each part substituted according to the same rule, before reassembling the parts, with the new string being added to the pool of possible products. Branching level and extension by poly-*N*-acetyllactosamine repeating units could be controlled by placing an optional limit on the total number of GlcNAc residues incorporated. Restrictions could be placed on individual enzyme activities by conditionals employing Boolean logic. The program could also be limited to use a subset of the enzymes. Simulations terminated after a prescribed number of iterations, or after any iteration in which no new products had been generated. The output of the program for three iterations of the method is shown in Fig 2. A web-application front end to the enzyme simulator (see Methods) is available online at http://www.boxer.tcd.ie/glycologue.

### Enzymes

The enzymes of Table 2 can be divided into five main classes of activity: initiation (**2**), core formation (**5**,**6**,**8**,**9**), branching and extension (**1**,**7**,**10**,**12**,**19**), sugar modification (**20**–**22**) and termination (**3**,**4**,**11**,**13**–**18**,**23**–**25**). The *terminal residue* of an oligosaccharide is the monosaccharide appearing at its non-reducing end. In the current model, the two methods of termination were fucosylation or sialylation of a terminal galactose. Sulfation was the only type of non-glycosyltransferase modification that was considered. Oligosaccharide chains can be of type 1 (ending in Gal*β*1-3GlcNAc-) or type 2 (ending in Gal*β*1-4GlcNAc-).

#### Initiation

*O*-Glycosylation is initiated by the transfer of a GalNAc to a free serine or threonine residue a nascent polypeptide, the reaction being catalysed by polypeptide *N*-acetylgalactosaminyltransferase. As many as 20 distinct ppGalNAc-T enzymes are encoded by the human genome, with 17 isoforms having been characterised to date [35, 36]. The isoforms are known to be differentially expressed, in different tissues, and to have different acceptor specificities [35]. Since the same reaction is catalysed by the different isoforms, they are treated in this work as a single entity.

#### Core formation

**Fig 3:**
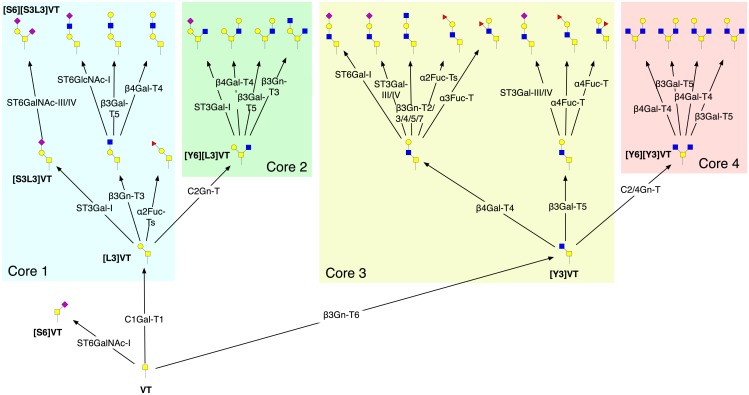
Initial stages of *O*-GalNAc glycosylation. Following the addition of GalNAc to an unoccupied serine/threonine residue on a polypeptide backbone, addition of Gal or GlcNAc forms cores 1–4, before further extension takes place. The structure identifiers shown are: VT (Tn); [S3L3]VT (ST); [S6]VT (STn); [S6][S3L3]VT (diST); [L3]VT (core 1); [Y6][L3]VT (core 2); [Y3]VT (core 3); [Y6][Y3]VT (core 4). Structures are displayed using CFG symbols. All reactions were predicted from four iterations of the method, with enzymes **1**–**18** of the model active. For reasons of space, not all reactions are shown.

Up to eight core structures can be formed by the addition of Gal, GalNAc or GlcNAc to the 3- and 6-linked positions of the GalNAc. We will be considering only the first four, which are the most commonly encountered: Gal*β*1-3GalNAc-Ser/Thr (core 1), GlcNAc*β*1-6[Gal*β*1-3]GalNAc-Ser/Thr (core 2), GlcNAc*β*1-3GalNAc-Ser/Thr (core 3) and GlcNAc*β*1-6[GlcNAc*β*1-3]GalNAc-Ser/Thr (core 4) [34]. Core 1 is formed by the enzyme C1Gal-T1 (**6**), which adds a *β*1,3-linked Gal from UDP-Gal to GalNAc. Core 1 formation can be followed by the actions of up to three enzymes with core-2 forming activity (**5**) to which we have assigned the short name C2Gn-T. Similarly core 3, formed by *β*3Gn-T3, can be modified to core 4 by C2/4Gn-T. The initial stages of *O*-glycosylation are depicted in Fig 3.

#### Extension and branching

*O*-Glycan branch length increases by the sequential addition of *N*-acetyllactosamine (LacNAc) residues through the alternating activities of *β*4Gal-T4 (**1**) and *β*3Gn-T2/3/4/5/7 (**10**), forming poly-LacNAc type-2 chains. These linear poly-*N*-acetyllactosamine glycans can be further branched by a *β*-l,6-*N*-acetylglucosaminyltransferase (Gcnt2; I-GnT) [37]. The activity of *β*4Gal-T4 is catalysed by up to six different isoforms [38], *β*4Gal-Ts 1 through 6, of which *β*4Gal-T4 is reported to be the dominant isoform in poly-*N*-acetyllactosamine chain extension of core-2 structures [39]. In the case of the I-branching enzyme, however, the isoform *β*4Gal-T1 is known to catalyse this reaction most efficiently [40]. For the activity of the I-branching enzyme itself, Gcnt2, we made two further assumptions based on the observations of Ujita *et al.*, (i) that Gcnt2 expects a terminal beta-1,4-linked galactose, described in this system by the pattern *[L4Y3L*; and (ii) that poly-*N*-acetyllactosamine extension by *β*3Gn-T2/3/4/5/7 is inhibited by the activity of the I-branching enzyme [40].

#### Modification

Both Gal and GlcNAc residues can be either 3-*O*- or 6-*O*-sulfated. We restricted the study to the Gal 3-*O*-sulfotransferase (GAL3ST2 and GAL3ST4) and GlcNAc 6-*O*-sulfotransferase (CHST4/6) activities. While there is evidence that sulfation is a late event during N-linked glycosylation [41], we assumed that sulfation can occur earlier in *O*-glycosylation, and that it does not preclude the actions of the other enzymes [10, 42].

#### Termination

*O*-Glycan branches can be terminated in a number of different ways that form important antigenic determinants, or epitopes. The principal structures are formed from the actions of various fucosyltransferases or sialyltransferases. The addition of either 3- or 4-linked fucose to the GlcNAc of a terminal LacNAc, can be followed by the addition of 2-linked fucose to the terminal Gal. A terminal galactose residue can be capped by either a 3-linked or 6-linked Neu5Ac, in the presence or absence of fucose. The ST3Gal-III isoform of enzyme **16** can also act on type-1 acceptors [43], according to the reaction pattern CMP-S + *[Lb3Y*T = CMP + *[Sa3Lb3Y*T. The A/B blood type and Sd^a^/Cad antigens are formed by the actions of enzymes **23**–**25**. The *β*4GalNAc-T enzyme (**25**) is active towards sialylated type-2 chains [44].

### Structure prediction

The enzyme rules were reversed, so that a single monosaccharide was removed at each step of the simulation. Any *O*-glycan structure supplied as an initial substrate to the reversed enzyme simulator was considered to be predictable, or deducible, within the system if its final step was the removal of the terminal GalNAc from the protein by the enzyme ppGalNAc-T, according to VT -- ppGalNAc-Ts --> T. If the simulation ended with no new products formed, and without reaching the non-glycosylated site, the glycan was considered non-predictable within the system.

### Reaction network generation

**Fig 4:**
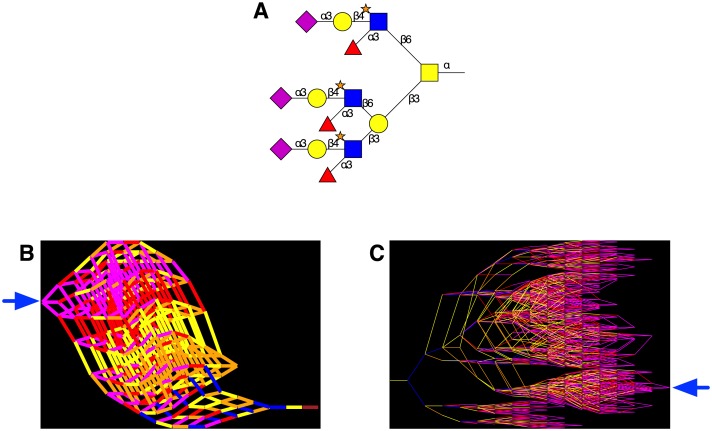
Simulated *O*-glycosylation reaction networks. **A** Graphical rendering of a 6-*O*-sulfated triantennary core-2 *O*-glycan with structure identifier [S3L4[f3][s6]Y6][[S3L4[f3][s6]Y6][S3L4[f3][s6]Y3]L3]VT. **B**. Predictive network in which the enzyme simulator is run in reverse, starting from the *O*-glycan structure identifier in (**A**), stopping when the final enzyme removes GalNAc from the protein. **C**. The reaction network generated in the forward (biosynthetic) direction using only the enzymes encountered in panel (**B**). Pathways are drawn from left to right. In (**B**) and (**C**), the structure drawn in panel (**A**) appears at the points indicated by the blue arrows. Nodes represent distinct *O*-glycans, and edges (reactions) are colour-coded by the type of monosaccharide being transferred: GalNAc (brown), Gal (yellow), Fuc (red), Neu5Ac (magenta), GlcNAc (blue) and sulfate (orange).

The reaction data provided by the method described earlier, and depicted in Fig 2, were used to generate network graphs in GraphViz (www.graphviz.org), with *O*-glycan identifiers as nodes and with edges representing enzyme-catalysed reactions, colour-coded according to the monosaccharide being transferred. The enzyme simulator also allowed enzymes to be knocked out *in silico*, either individually or in groups, with each knockout resulting in a different reaction network. A web application, O-Glycologue (see Methods) was developed in order to view the structures obtained for a particular set of knockouts, and compare them with the structures obtained for the “wild-type” network, defined as the network obtained with all 25 of the enzymes active. The method is illustrated with an example taken from a study on salivary MUC7 glycans [45], a triantennary core-2 structure with the structure identifier [S3L4[f3][s6]Y6][[S3L4[f3][s6]Y6][S3L4[f3][s6]Y3]L3]VT (Fig 4A). The reversed reaction network is shown in Fig 4B, which successfully removed all monosaccharides in 17 iterations using the nine enzyme activities **1**, **2**, **5**–**7**, **11**, **16**, **19** and **20**. The network of reactions produced when the enzyme simulator was run in the forward direction with only these enzymes active is shown in Fig 4C.

### Network properties

With all 25 enzyme activities enabled, 18 iterations of the method generated 13,127,561 unique *O*-glycans, in 34,215,049 reactions. All structure identifiers generated by the enzyme simulations were shown to be valid according to the parser. Different epitopes could be determined from the terminal sequences of the identifier string, and were counted as percentages of the total number of glycans formed: Lewis A ([L3[f4]Y, 13.2%), Lewis X ([L4[f3]Y, 25.0%), sialyl-Lewis A ([S3L3[f4]Y, 4.2%), sialyl-Lewis X ([S3L4[f3]Y, 8.4%), Lewis B ([[f2]L3[f4]Y, 4.3%), Lewis Y ([[f2]L4[f3]Y, 8.2%), H antigen ([[f2]L3Y, 9.4%), A ([V3[f2]L3[f4]Y, 1.9%), B ([La3[f2]L, 17.5%), Sd^a^/Cad ([S3[Vb4]L, 12.7%) and other (24.7%).

Depending on the degree of branching, several different epitopes could appear together on the same *O*-glycan. Overall, 227 different pattern combinations of recognised epitopes could be distinguished, such as Lewis A with the H antigen.

**Fig 5:**
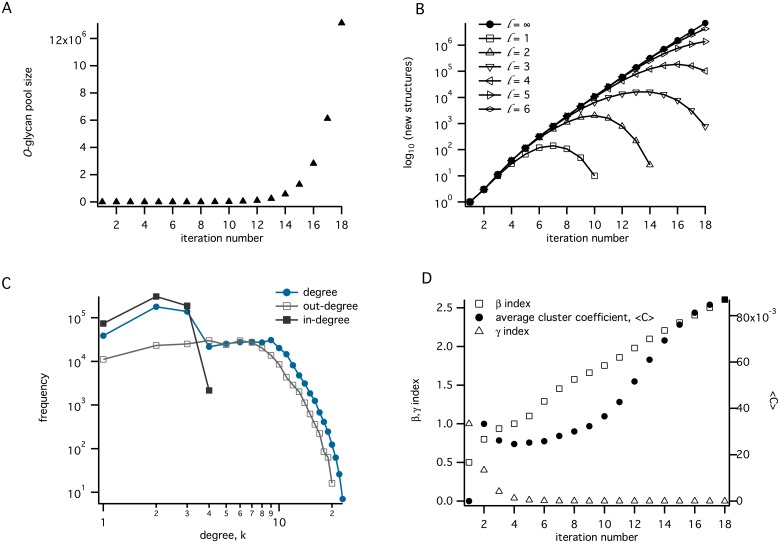
Network properties. A. The total number of *O*-glycans produced as a function of iteration number. B. The number of new structures appearing at each iteration number, for a series of networks limited by the maximum number of GlcNAcs incorporated (*l*), as indicated. C. The degree distribution after 14 iterations. D. Variation of *β* and *γ* indices, and network average clustering coefficient (〈*C*〉) with increasing iteration number.

As a consequence of the method used to produce the network, in which the products at iteration *n* + 1 are dependent only upon those arising from iteration *n*, the growth function can be approximated by a discrete logistic map, *ν*(*n* + 1) = *bν*(*n*), *b* > 1, with solution *ν*(*n*) = *ab*^*n*^. Although the total population is therefore expected to grow exponentially, by setting a limit on the maximum number of GlcNAc residues incorporated in each *O*-glycan, it was possible to close the networks, so that eventually no further structures were added to the glycan pool (Fig 5B).

Under the assumption of irreversibility of each reaction, the network can be viewed as a rooted, directed acyclic graph *G* = (*V*,*E*), where *V* and *E* are sets of nodes and edges, respectively, with each node representing a distinct *O*-glycan and edges representing enzyme-catalysed reactions in which *O*-glycans appear as substrates or products. The *degree* of a node is defined as the number of its immediate neighbours to which it is connected by an edge. For a directed graph, the number of incoming nodes is called the in-degree, and the number of outgoing nodes is defined as the out-degree. An important network property is the *degree distribution*, which is frequently expressed in terms of the probability, *P*(*k*), that a randomly selected node will be of degree *k*. Many real networks possess the property of hierachical clustering of nodes [46] with a degree distribution that displays a power-law tail, *P*(*k*)∼*k*^ − *λ*^. In contrast, our reaction network displayed a Poisson-like distribution that is characteristic of random networks [47]. After 14 iterations, the average degree of the network, 〈*k*〉, was calculated to be 4.36, with the in-degree and out-degree averages each equal, at half of this value. A bilog plot of the degree-distribution of the network (node degree frequency vs degree) is non-linear, as shown in Fig 5C, indicating that the network is not self-similar [48], or scale-invariant. That the degree distribution of a reaction network arising from a fully deterministic system has the characteristics of a random network may be a natural outcome of the method that was used to generate the glycan structure libraries. Since this method is essentially combinatoric, in that every possible acceptor-product is discovered from every substrate, we conjecture that its degree distribution can be described by a binomial function. Newman *et al.*[49] have shown that networks with a binomial degree distribution become Poisson when the number of nodes is large.

Quantitative measures of the connectedness of the reaction network are provided by the *α*, *β* and *γ* indices [50]. The *β* index is the ratio of the number of edges, *e*, to the number of nodes, *v*:

$$\begin{array}{c} \beta =\frac{e}{v} \end{array}$$ (1)

The definitions of the non-planar versions of the *α* and *γ* indices, which allow for edges to cross at non-nodal positions in the plane, are

$$\begin{array}{c} \alpha =\frac{\left(e-v\right)}{v(v-1)/2-(v-1)} \end{array}$$ (2)

and

$$\begin{array}{c} \gamma =\frac{2e}{v(v-1)}. \end{array}$$ (3)

The *α* index represents the number of cycles in a graph to the maximum number of possible cycles, and will take a value between 0 and 1. The *γ* index is the ratio of the number of edges to the total number of edges in the fully connected network, *v*(*v* − 1). Local clustering coefficients were also computed, and averaged across the complete reaction network [51]. The clustering coefficient, *C*_*i*_, provides a measure of the fractional degree to which nearest neighbours of node *i* are connected to each other. Let *k*_*i*_ be the number of immediate neighbours of node *i*. Since there can be at most *k*_*i*_(*k*_*i*_ − 1) edges between *k*_*i*_ nodes, for a directed graph, the value of *C*_*i*_ is defined as

$$\begin{array}{c} C_{i}=\frac{E_{i}}{k_{i}\left(k_{i}-1\right)} \end{array}$$ (4)

where *E*_*i*_ is the number of existing edges between the neighbours of node *i*. An average network clustering coefficient, 〈*C*〉, was defined over the whole reaction network. The values of *β* and 〈*C*〉, which were calculated at each iteration of the enzyme simulation, showed an increase overall, monotonically above the iteration 7, while the non-planar *γ* index decayed uniformly from unity (Fig 5D). The increase in *β* index approximated to linearity above iteration 8.

### Enzyme knockouts

**Fig 6:**
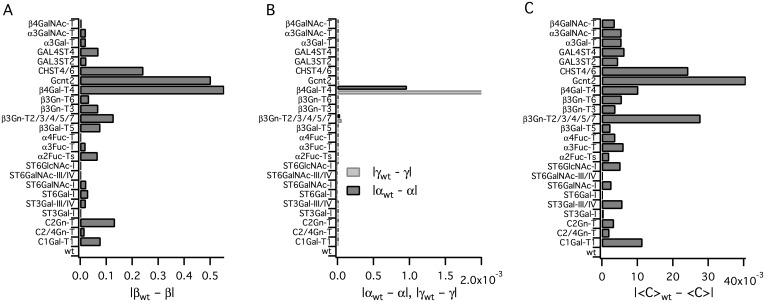
*In-silico* enzyme knockouts. Effects of *in-silico* enzyme knockouts on network indices. The effects of single-enzyme knockouts on (A) the *β* index, (B) *α* and *γ* indices and (C) the network average clustering coefficient 〈*C*〉 are shown; each network in A–C was generated using 15 iterations of the method described in the text; the ordinate axis in each case shows the name of the enzyme being knocked out, while the abscissa shows the difference between the wild type and knockout indices.

We simulated the effects of knocking out individual enzymes, observing the changes incurred in the topology of this reaction network. *O*-Glycan heterogeneity was most strongly influenced by the activities of Gcnt2, C2/4Gn-T, *β*3Gn-T2/3/4/5/7, *β*3Gn-T6 and *β*4Gal-T4, as quantified by the changes in the indices in Fig 6A–6C. Changes to local clustering coefficients were also noticeable, although they were not as marked. In the absence of enzyme *β*3Gn-T2/3/4/5/7 (**10**), the network closed after 20 iterations, and in the absence of *β*4Gal-T4 (**1**), the network was closed after 14 iterations, since no further extension of antennae was possible in the absence of either of these activities. Changes to the *α* and *γ* indices were notable only for these two enzymes (Fig 6B).

Changes to the distributions of common epitopes are given in Table 3. The occurrences of each epitope, expressed as a percentage of the total number of unique *O*-glycans, were obtained for 12-iteration networks with the enzyme knocked out as indicated, and from which the sulfotransferases (**20**–**22**) had been omitted. Excluded from the results are ppGalNAc-Ts and the knockouts of the sialyltransferases **17** and **18**, which showed no alteration from “wild type” (wt). Since more than one epitope can be expressed on a single *O*-glycan, the numbers on each line in the table need not sum to 100. The *β*4Gal-T4 knockout was found to eliminate all glycans expressing Le^x^, SLe^x^, Le^y^ and Sd^a^ antigens, indicating that it is an essential component of their biosynthesis; an increase in the percentage of *O*-glycans bearing the B antigen was also observed. The greatest decrease in the total number of glycans formed was observed with this knockout (not shown). Single-enzyme knockouts of the *N*-acetylglucosaminyltransferases did not affect the distributions of these epitopes so markedly, as might be expected from their functions in core formation, elongation and branching, rather than termination. Knocking out the *β*-1,3-galactosyltransferase activity eliminated only *O*-glycans expressing the B antigen.

**Table 3:** Effects of single-enzyme knockouts on the distributions of common epitopes. The numbers of *O*-glycans are expressed as percentages of the total number of glycans obtained in each experiment. See text for details.

	Knockout	Le^a^	Le^x^	SLe^a^	SLe^x^	Le^b^	Le^y^	H	A	B	Sd^a^	other
	wt	8.7	15.0	4.1	7.0	4.1	6.9	8.7	1.9	16.0	10.5	32.8
**1**	*β*4Gal-T4	14.2	0.0	14.2	0.0	14.2	0.0	14.2	14.2	30.3	0.0	22.5
**3**	*α*4Fuc-T	0.0	16.3	0.0	7.8	0.0	7.7	9.8	0.0	16.0	11.6	41.1
**4**	*α*2Fuc-Ts	12.6	21.2	6.4	11.1	0.0	0.0	0.0	0.0	0.0	17.2	41.2
**5**	C2Gn-T	8.7	15.0	4.1	7.0	4.1	6.9	8.7	1.9	16.0	10.5	32.8
**6**	C1Gal-T1	8.7	14.5	4.6	7.4	4.6	7.2	8.7	2.4	17.2	11.3	30.2
**7**	*β*3Gn-T3	8.2	14.2	4.3	7.3	4.3	7.1	8.2	2.3	18.2	11.1	31.0
**8**	*β*3Gn-T6	8.7	15.2	3.8	6.8	3.8	6.7	8.7	1.6	15.5	10.1	34.1
**9**	C2/4Gn-T	8.5	15.0	3.8	7.0	3.8	6.8	8.5	1.6	15.7	10.4	34.0
**10**	*β*3Gn-T2/3/4/5/7s	8.7	15.0	4.1	7.0	4.1	6.9	8.7	1.9	16.0	10.5	32.8
**11**	*α*3Fuc-T	10.6	0.0	5.3	0.0	5.3	0.0	10.6	2.6	17.5	10.1	45.5
**12**	*β*3Gal-T5	0.0	19.5	0.0	9.7	0.0	9.7	0.0	0.0	14.8	14.0	41.5
**13**	ST6Gal-I	9.4	16.4	4.5	7.9	4.5	7.7	9.4	2.1	18.0	11.9	27.4
**15**	ST3Gal-I	8.7	15.0	4.1	7.0	4.1	6.9	8.7	1.9	16.1	10.5	32.7
**16**	ST3Gal-III/IV	10.8	18.4	0.0	0.0	5.3	9.0	10.8	2.6	21.0	0.0	35.7
**19**	Gcnt2	8.6	8.6	6.3	6.4	6.3	6.3	8.6	4.3	21.4	11.1	29.3
**23**	*α*3Gal-T	9.4	16.2	4.5	7.8	4.5	7.6	9.4	2.1	0.0	11.7	39.1
**24**	*α*3GalNAc-T	9.4	16.2	4.5	7.8	4.5	7.6	9.4	0.0	17.7	11.7	28.9
**25**	*β*4GalNAc-T	9.1	15.8	4.3	7.4	4.3	7.4	9.1	2.0	17.2	0.0	36.7

### Structure validation

The predictive power of the enzyme simulator was tested by comparing the *in-silico* generated *O*-glycans against fifteen published collections of such structures that had been identified experimentally: mucin *O*-glycans from human colon [52, 53]; structures of MUC1 mucin glycoforms obtained from normal and cancerous breast epithelial cell lines [54]; poly*-N-*acetyllactosamine extended structures of leukosialin glycoprotein obtained from promyelocytic and myelogenous leukaemia cell lines [55]; leukosialin *O*-glycans expressed in T-lymphocytic leukemia [56] and erythroid, myeloid, and T-lymphoid cell lines [57]; *O*-glycans from salivary MUC7, a major component of mucin glycoprotein 2 (MG2) [45]; *O*-glycans of Tamm-Horsfall glycoprotein [58]; sulfated core-2 and core-4 oligosaccharides obtained from mucins associated with chronic bronchitis [59]; bovine serum fetuin, human serum IgA1 and secretory IgA, human neutrophil gelatinase B and glycophorin A *O*-glycans [60]; extended core-1 and core-2 *O*-glycans from Chinese hamster ovary (CHO) cells transfected with *β*3Gn-T3 [61]; MUC1 and MUC4 *O*-glycans from bovine and human milk [62], normal human serum [63] and a human gastric adenocarcinoma cell line (MKN45) [64]; mucin from normal descending colon [65]; recombinant mucins from engineered CHO cells [66]. In all, 244 unique *O*-glycan structures were collected from these studies and assigned structure identifiers. Multiple identifiers were assigned where a number of different configurations was possible. For example, the monosialylated forms of Gal*β*1-3(Gal*β*1-4GlcNAc*β*1-6)GalNAc-R [64] were represented by the separate identifiers [L4Y6][S3L3]VT and [S3L4Y6][L3]VT.

Each member of the set of experimentally determined *O*-glycans was supplied to the reversed enzyme simulator as the starting substrate, and tested for predictability within the system. Overall, 87% of the unique *O*-glycan structures were predicted by the method, which was able to reproduce any of the extended branched core 1–4 structures, with sialyl-Lewis X, Lewis Y, Lewis A or -B terminals and their 3′- and 6-sulfated variants. Table 4 lists the *O*-glycans determined experimentally that appeared in more than one of the studies, and thus independently verified, in descending order of frequency. Shown are the structure identifier, the supporting literature and a check next to those structures that were predicted *in silico*. Of the 45 oligosaccharides most commonly occurring, 44 were predicted by the model, giving a coverage of 98%.

**Table 4:** *O*-Glycans common to more than one published study, with their predictions *in silico*. The structure marked NP was not predicted by the model constructed from the unmodified activities of Table 2. The sources of each glycan are given as reference numbers.

Structure identifier	Sources	*In silico*
[S3L3]VT	[45, 54–57, 60–64, 66]	✓
[S6][S3L3]VT	[45, 54–57, 60–63, 66]	✓
[S3L4Y6][S3L3]VT	[45, 54–57, 60, 61, 63, 64, 66]	✓
[S3L4Y6][L3]VT	[45, 55–57, 60–64, 66]	✓
[L4Y6][S3L3]VT	[45, 55–57, 60–64, 66]	✓
[S6][L3]VT	[45, 56, 57, 60, 62, 63, 65, 66]	✓
[L4Y6][L3]VT	[45, 55, 58, 60, 62–64, 66]	✓
[L3]VT	[45, 54, 55, 57, 60, 63, 66]	✓
[Y6][S3L3]VT	[60–64, 66]	✓
[S6]VT	[52, 53, 57, 63, 65]	✓
[L4[f3]Y6][S3L3]VT	[45, 58, 60, 62, 63]	✓
[S3L4[f3]Y6][S3L3]VT	[45, 58, 61, 63]	✓
[S3L4[f3]Y6][L3]VT	[45, 58, 60, 63]	✓
[L4Y3L4Y6][S3L3]VT	[60, 62, 64, 66]	✓
VT	[54, 55, 57, 60]	✓
[Y6][L3]VT	[60, 62, 66]	✓
[S6][Y3]VT	[52, 53, 65]	✓
[S6][L4Y3]VT	[52, 53, 65]	✓
[L4[f3]Y6][L3]VT	[45, 60, 62]	✓
[L4Y3L4Y6][L3]VT	[60, 62, 64]	✓
[Y3]VT	[52, 66]	✓
[Y3L4Y6][L3]VT	[60, 64]	✓
[S6][S6L4Y3]VT	[52, 53]	✓
[S6L4Y6][S3L3]VT	[60, 66]	✓
[S6L4Y3L4Y6][S3L3]VT	[60, 66]	✓
[S3L4[f3]Y6][[S3L4[f3]Y6][S3L4[f3]Y3]L3]VT	[45, 58]	✓
[S3L4[f3]Y6][[S3L4[f3]Y6][L4[f3]Y3]L3]VT	[45, 58]	✓
[S3L4[f3]Y6][[S3L4Y6][S3L4[f3]Y3]L3]VT	[45, 58]	✓
[S3L4[f3]Y6][[L4[f3]Y6][S3L4[f3]Y3]L3]VT	[45, 58]	✓
[S3L4[f3]Y6][[L4[f3]Y6][S3L4Y3]L3]VT	[45, 58]	✓
[S3L4[f3]Y6][[L4Y6][S3L4[f3]Y3]L3]VT	[45, 58]	✓
[S3L4Y6][[S3L4[f3]Y6][S3L4[f3]Y3]L3]VT	[45, 58]	✓
[S3L4Y6][[L4[f3]Y6][S3L4[f3]Y3]L3]VT	[45, 58]	✓
[S3L4Y3]VT	[65, 66]	✓
[S3L4Y3L4Y6][S3L3]VT	[64, 66]	✓
[S3L4Y3L3]VT	[61, 66]	✓
[L4[s6]Y6][S3L3]VT	[63, 66]	✓
[L4[f3]Y6][[S3L4[f3]Y6][S3L4[f3]Y3]L3]VT	[45, 58]	✓
[L4[f3]Y3L4Y6][S3L3]VT	[60, 62]	✓
[L4Y6][[L4Y6][L4Y3]L3]VT	[45, 62]	✓
[L4Y3]VT	[52, 66]	✓
[L4Y3L4[f3]Y6][L3]VT	[60, 62]	NP
[L4Y3L4Y3]VT	[52, 66]	✓
[L4Y3L4Y3L4Y6][L3]VT	[60, 64]	✓
[L4Y3L3]VT	[61, 66]	✓

## Discussion

From analysis of the grammar, and the results of the enzyme simulations, we predict that a highly heterogeneous population of mucin *O*-glycans is likely to result if even a limited subset of the enzyme activities of Table 2 is expressed. *In-silico* enzyme knockouts have identified *β*4Gal-T4 as a key regulator of the complexity of *O*-glycosylation networks, in keeping with our earlier observations on the influence of this enzyme on N-linked glycosylation in engineered Chinese hamster ovary cells [67].

The number of iterations was chosen according to the type of *in-silico* experiment: trends in the changes to the indices were discernable by iteration 15, hence this value was chosen for the enzyme-knockout studies; 18 is the maximum number of iterations of the basic model that were possible within the available memory (32 GB), with all 25 enzymes active and no limitations placed on the number of GlcNAcs. Not all of the enzymes in the current model will be present in all species, or active at all times. The full network is therefore a chimeric construct, but one which could be tailored for specific cases as needed, by considering only the enzymes known to be expressed in a particular organism or tissue. The O-Glycologue web application, described in Methods, provides an easy way to experiment with the effects of knockouts or knock-ins of the enzymes of *O*-glycosylation.

The transferase activities leading to cores 5 through 8 are as yet uncharacterized [1], but could be added in future to account for such structures as are occasionally found in colonic tissues. The *O*-glycan structure [L4Y3L4[f3]Y6][L3]VT was also not predicted by the current model (Table 4). Although its appearance could be the result of a wider acceptor specificity of *β*3Gn-T2/3/4/5/7 (**10**) that would allow this enzyme to act according to the pattern *[Lb4[fa3]Y*T, it could also be the result of fucosylation of an inner GlcNAc by one of the several known *α*1,3-fucosyltransferase variants, such as FUT4 [68]. The pattern corresponding to the substrate acceptor in such a case would be *Lb4Y*T. An additional *α*1,3-fucosylation pattern that was evident from this data set is the sequence *L4[f3]Y6*, evident in ten of the non-predicted glycans from two studies [60, 62], and in the sole non-predicted structure of Table 4. It is likely that a fucosyltransferase activity exists that is yet to be characterized, and which acts on type-2 chains with a preference for the 6-linked GlcNAc of core-2 or core-4 *O*-glycans. In the future, these reactions, as well as those of other fucosyltranserases that are distinguished by different substrate specificities, could be incorporated into the simulator either as additional rules or as refinements of the existing rule (**11**).

Some structures that were not predicted may also have been mischaracterised. For example, the non-predicted glycan structure described by Podolsky [52], to which we assigned the identifier [S6][[S6L3Y6][S6L3Y3]L4Y3]VT, is in the same paper identified as a type-2 structure, which could be predicted. Our validation study therefore provides a lower bound on the number of structures that can be predicted. Certain poly-6-sialylated structures, including [S6][S6L3Y3[S6]L4Y3]VT, were not predicted. It is possible that a sialyltransferase activity exists in colon that recognises galactose at a distance from the non-reducing end of an oligosaccharide; for instance, an alternative reaction of ST6GlcNAc-I (**18**) might be CMP-S + *Y3Lb4Y*T = CMP + *Y3[Sa6]Lb4Y*T.

Our analysis of the monosaccharide content of *O*-glycans extracted from the CFG database revealed that the frequency of occurrence of Neu5Ac was between two and three times the total of the remaining monosaccharides of lesser occurrence: Glc, GlcA, Kdn, and Neu5Gc. Of these, Neu5Gc, or *N*-glycolylneuraminic acid, is of particular interest because it is immunogenic in humans as a result of the silencing of CMP-*N*-acetylneuraminate monooxygenase (EC 1.14.18.2). This enzyme, which is active in other mammalian species, adds a single oxygen to CMP-*N*-acetylneuraminate to form CMP-*N*-glycolylneuraminate. Neu5Gc obtained in the diet can become incorporated into the cell surface glycome, especially that of cancerous tissue, making it a potential target for immunotherapy [69]. Sialic acids entering the cell via endocytic pathways become activated by the nuclear enzyme CMP-sialate synthase (EC 2.7.7.43, *N*-acylneuraminate cytidylyltransferase) [70]. Together with the observation that CMP-Neu5Gc can readily substitute for the native donor in reactions catalysed by the sialyltransferases from other species [71], a reasonable assumption is that Neu5Gc is incorporated into human glycoforms by this means. Thus, while Neu5Ac may be the dominant component of the sialylated epitopes expressed in O-linked and N-linked glycoproteins, a portion of such glycans generated by the enzyme simulator could be considered as terminating in Neu5Gc. If the sialyltransferase activities of Table 2 were allowed to act with CMP-Kdn as donor, an additional six structures from the validation study could be predicted by the model, increasing coverage of the data set to 89%.

The notation we have described provides a succinct way to encode structural information for both graphical representation and modelling. Other linear string representations of carbohydrates exist, such as LINUCS [72] and Linear Code [31], which are broader in scope than *O*-GalNAc glycosylation, and are supported by established glycoinformatic software tools, such as GlycoWorkbench [73]. An advantage of the modelling language described in this work is that it is able to encode the sialic acid Neu5Gc, which cannot be expressed in Linear Code. A more general, and widely supported carbohydrate encoding format is GlycoCT [32]. More recently, the Web3 Unique Representation of Carbohydrate Structures (WURCS) formalism was introduced with an even wider scope [74]. The GlycoForm web application, described in the methods, is able to output any *O*-glycan structure identifier as both IUPAC, Linear Code and GlycoCT condensed formats, making it interoperable with other software and databases. For the purposes of modelling and display, however, the advantages of the structure identifiers presented in this work are twofold; first, adherence to a strictly one-letter system for the monosaccharides reduces the memory requirements, which can be large when all enzymes of the model are allowed to act; second, the lexical analysis is simplified, since in the drawing algorithm each character can act as a single instruction.

The method could be adapted to other systems, depending on the intended application. For instance, other enzyme activities could be included to account for branch termination by *α*-GlcNAc, as has been observed in porcine gastric mucins [10], but not commonly on human glycoproteins [42]. The formal grammar could be modified to describe *N*-glycans, such as those expressed on immunoglobulins [75], the hypermannosylated glycans produced by yeasts [76], or glycans initiated through O-linked fucose [77] or mannose [78]. Additional reaction rules could be supplied, as needed, to support the enzyme activities of galactose 6-*O*-sulfotransferase and *α*-2,8-sialyltransferase. A limitation of the current implementation is that not all routes to a product may be included: for example, the simulated activity of Core-2 forming enzyme (**5**) does not recognise a 3-linked sialic acid on the lower arm of Core 1. The alternative route to [Y6][S3L3]VT could be accommodated by including sialic acid as an option to the reaction pattern, similar to the case for reactions that allow sulfation of Gal or GlcNAc.

Although we have restricted our subject to the enzymes of *O*-glycan biosynthesis, the actions of glycosidases, which are involved in *O*-glycan degradation, may have an important regulatory role. For example, it is known that *α*-l-fucosidase (EC 3.2.1.51) is downregulated in certain types of colorectal cancer [79], from which we infer that an increase in Lewis-type epitopes might be the result of both increased fucosyltransferase activity in Golgi and decreased fucosidase activity in either tissue or plasma. In the future, therefore, this model could be extended to include enzymes involved in the catabolism of O-linked glycoproteins. A quantitative analysis of O-linked glycosylation, incorporating the kinetic parameters of the enzymes involved, would be a natural extension, and development along these lines is proceeding.

The web application, O-Glycologue, provides a convenient way to draw *O*-glycan structures from the identifiers used in this work, and to explore the wide variety of possible oligosaccharide structures formed by the activities of several known enzymes of *O*-glycosylation. While a MATLAB-based system for modelling N- and O-linked glycosylation has recently appeared [15], the system described in this article requires neither installation by the user nor a commercial software license. To our knowledge, O-Glycologue is the first tool capable of testing the effects of knockouts of the enzymes of O-linked glycosylation on glycoform heterogeneity. As a knowledge-based system, it should be useful to glycobiologists interested in predicting the biosynthetic pathways forming particular *O*-glycans. Given that the glycoslation of mucins is known to change during cancer progression [7, 69], the software may be an aid to discovering the enzyme activities most responsible for the formation of particular cancer biomarkers.

In conclusion, we have presented a method for encoding and displaying mucin-type *O*-glycans, and a method for generating reaction networks from enzymes known to act in *O*-glycosylation. The formal grammar and the enzyme reaction rules of Table 2, together with an initial glycan identifier as an axiom, comprise the deductive apparatus of a formal system for the modelling and display of these *O*-glycans. Through an analysis of the reaction networks, we predict that *β*4Gal-T4 is a key regulator of mucin-type *O*-glycan heterogeneity, along with *β*3Gn-T2/3/4/5/7, Gcnt2, C1Gal-T, C2Gn-T and CHST4/6. A comparison of the output of the model with experimentally derived glycans suggests the existence of several novel activities. This approach, which has been validated by structure predictions and the effects of enzyme removal, is intended to form a basis for future kinetic evaluations, and extensions to accommodate other types of glycan structure.

## Supporting Information

S1 Text Enzyme simulator source. Source code of the enzyme simulator written in Python 3.
(TGZ)

S2 Text Structure identifiers used in validation studies. (TXT)
